# Continuous visual stimulus tracking to quantify eye motility in spinocerebellar ataxia type 3

**DOI:** 10.3389/fneur.2025.1650269

**Published:** 2025-12-15

**Authors:** M. J. de Boer, R. A. Wasmann, J. W. R. Pott, F. W. Cornelissen, N. M. Jansonius

**Affiliations:** 1 Laboratory of Experimental Ophthalmology, University Medical Center Groningen, University of Groningen, Groningen, Netherlands; 2 Department of Ophthalmology, University Medical Center Groningen, University of Groningen, Groningen, Netherlands

**Keywords:** spinocerebellar ataxia, SCA3, eye-tracking, eye movements, continuous visual stimulus tracking, disease severity, biomarker

## Abstract

**Introduction:**

Spinocerebellar ataxias (SCA) form a group of dominantly inherited neurodegenerative diseases represented by progressive cerebellar ataxia and various other neurological deficits. SCA3 is the most prevalent type globally and represents 28% of the autosomal dominant cerebellar ataxias in The Netherlands. The associated oculomotor disorders, with distance esotropia as its hallmark, cause diplopia and often present early. To gain further insight into this, we examined eye movements made during a continuous visual stimulus tracking task (SONDA; Standardized Oculomotor and Neuro-Ophthalmic Disorder Assessment).

**Methods:**

Thirteen genetically confirmed SCA3 cases underwent SONDA, both monocularly and binocularly. As a reference, we used previously collected data from 36 monocularly and 13 binocularly measured healthy subjects.

**Results:**

SCA3 cases were well capable of tracking the moving stimulus, but they performed the task differently. More specifically, their eyes were not synchronized in their movements, and they made multiple small saccades in response to a large stimulus jump instead of a larger saccade followed by a small corrective saccade. The saccadic amplitude distribution shape was related to the severity of the oculomotor disorder, suggesting that the saccadic amplitude distribution could be used as a biomarker of disease severity.

**Conclusion:**

Overall, this study highlights that eye-tracking during a standardized task can give valuable insights into how eye movements are affected in SCA3 and provides suggestions for potential biomarkers for severity and the associated treatment options. Longitudinal research is needed to elaborate on these findings and validate the proposed biomarkers.

## 1 Introduction

Spinocerebellar ataxias (SCA) constitute a group of dominantly inherited neurodegenerative diseases characterized by progressive cerebellar ataxia and other neurological deficits. SCA type 3 (SCA3), the most prevalent type globally, accounts for 28% of the autosomal dominant cerebellar ataxias in Netherlands (1). This condition arises from an unstable polyglutamine-encoding CAG repeat expansion in the ATXN3 gene, leading to neuronal toxicity and cerebellar ataxia with associated oculomotor disorders. Coordination and maintaining proper alignment of the eyes are essential for accurately perceiving one single image and depth perception. This process, known as sensory fusion, occurs when the cortex receives and combines these two images into one. When the cortex is unable to achieve sensory fusion, extraocular vergence movements bring the images within the fusion area. When this system is affected, it can result in strabismus and diplopia. One of the characteristics of SCA3 is progressive esotropia. Approximately 80% of the patients with SCA3 experience diplopia (2). Eventually, the disorder progresses, leading to more severe oculomotor abnormalities like an impaired smooth pursuit and slowing of saccades (3, 4). High saccade velocities enhance vision by shortening saccade duration and minimizing the period of visual blur caused by image slip across the retina. A reduction in saccade accuracy or velocity negatively impacts vision.

Currently, no causal therapy exists to prevent or slow the progression of SCA3. However, symptomatic treatment strategies are available for progressive esotropia. These treatments typically involve the use of prism-corrected glasses, with increasing prism power over time. When prism correction is no longer possible, surgical strabismus correction is considered. Eventually, treatment can no longer restore binocular single vision. To optimize treatment, it is crucial to gain a deeper understanding of how eye movements are affected in SCA3, and how they can be quantified. Previous studies have demonstrated the value of the Standardized Oculomotor and Neuro-Ophthalmic Disorder Assessment (SONDA) (5) in providing information on the integrity of the visual field in glaucoma (6) and on oculomotor control in other neurological disorders (5, 7). These findings suggest that SONDA could be a powerful tool in the assessment of SCA3.

The aim of this study was to gain a deeper understanding of how eye movements are affected in SCA3 and how they can be quantified. This understanding is crucial for monitoring disease progression and ultimately optimizing treatment strategies for the progressive esotropia associated with SCA3. For this purpose, we performed SONDA in a group of SCA3 patients with a wide range of disease severity and related the findings to data collected in healthy subjects.

## 2 Materials and methods

### 2.1 Participants

Thirteen patients with genetically confirmed SCA3 (cases) underwent oculomotor assessments using SONDA. As a reference, we used previously collected SONDA data from 36 healthy subjects with measurements under monocular conditions (monocular controls) and 13 with measurements under binocular conditions (binocular controls). The study followed the tenets of the Declaration of Helsinki. This study was approved by the local Ethics Committee (METc 2020/472). Written informed consent for participation was obtained from all participants.

### 2.2 Recruitment and screening

All SCA3 patients that received treatment in our hospital in 2022 or 2023 were invited to participate, and those who provided informed consent were included in this study. All patients had normal or corrected-to-normal visual acuity better than 6/12 (below 0.3 logMAR). We excluded patients that were unable to perform SONDA, had other ophthalmic abnormalities, or never had binocular single vision. Ophthalmic and neurological data were obtained from the electronic health record system.

Controls were recruited as part of an earlier study (6). In short, potential controls who responded to an advertisement were asked to fill out a questionnaire that screens for known eye or neurological abnormalities, or a positive family history of glaucoma. Hereafter, a short eye-health screening was performed, which included measurements of the intraocular pressure (IOP; Ocular Response Analyzer G3 non-contact tonometer, Reichert Technologies, Inc., Depew, NY, United States) and the best-corrected visual acuity (BCVA; Nidek ARK-1 s. Nidek co., ltd, Gamagori, Japan), frequency doubling technology (FDT) perimetry (C20-1 screening mode; Carl Zeiss, Jena, Germany), and an assessment of the peripapillary retinal nerve fiber layer thickness with Spectral Domain Optical Coherence Tomography (SDOCT, Copernicus, Optopol Technologies, Zawierci, Poland). Exclusion criteria for controls were any known eye abnormality, a positive family history of glaucoma, neurological disorders that could affect test performance, a BCVA above 0.1 logMAR, an IOP above 22 mmHg, a reproducibly abnormal test location on the FDT test result, or any temporally, inferiorly, or superiorly located red clock hour abnormality in the peripapillary retinal nerve fiber layer on the OCT.

### 2.3 Experimental set-up

Participants were seated in front of a 24.5-inch IPS monitor (OptixMag251RX, Micro-Star International Co., Ltd., New Taipei City, Taiwan) at a viewing distance of 60 cm, resulting in a visual angle of 49 × 29 degrees. Their head was placed in a chin- and forehead rest to minimize head movements. Because some cases were in a wheelchair, it was not always possible to use the chin- and forehead rest, and head positioning was not always optimal. The experiment was performed in a dark and quiet room; the only illumination present was provided by the monitor.

The monitor had a resolution of 1920 × 1080 pixels and a frame rate of 240 Hz. Monitor luminance was calibrated as described in Vrijling and de Boer et al. (8). For eye movement recording, we used an Eyelink Portable Duo (SR Research Ltd., Ottawa, Ontario, Canada), running software version 6.10.01. Stimulus display and eye movement recording were controlled by the Psychtoolbox and Eyelink Toolbox extensions (9–12) for MATLAB (The Mathworks, Inc., version 2021a). We used an HP Elitedesk 800 G3 with a NVIDIA Geforce GTX 1080 graphics card, running Windows 10 as the host PC for stimulus display.

Eye movements were recorded at a sampling frequency of 1,000 Hz. The eye-tracker was calibrated at the start of the experiment using the built-in 9-point calibration routine for controls and the built-in 5-point calibration routine for cases. We chose the shorter 5-point calibration routine for cases as it was known that most of them would have difficulty with stable fixation. Calibration was directly verified with the validation procedure in which the same nine/five points were displayed again. For controls, the experiment was only started if the calibration accuracy was sufficient (i.e., average error of less than 0.5° and a maximum error of less than 1.5°). If one of the nine points could not be recorded accurately (i.e., error > 1.5°; this mostly occurred in one of the upper corners), while the remaining eight points only had a small error, the experiment was started regardless. For the cases, the validation procedure was performed once, after which the experiment was started regardless of calibration accuracy.

### 2.4 Standardized oculomotor and neuro-ophthalmic disorder assessment (SONDA)

The SONDA stimulus used in the current experiment consisted of a moving Gaussian blob presented on a static gray background of 30 cd/m^2^ at either a Weber contrast of 640% (for cases and monocular controls) or 160% (for binocular controls). For these contrasts, tracking performance has reached a ceiling level in (older) controls [see Vrijling and de Boer et al. (6)], indicating that the use of different stimulus contrasts (due to the re-use of data for the controls) was unlikely to impact the conclusions from the current study. The blob’s diameter was 0.43 degrees of visual angle, corresponding to the diameter of a Goldmann size III stimulus, which is commonly used in visual field testing. The participant was asked to follow the moving stimulus with their eyes.

**Figure 1:**
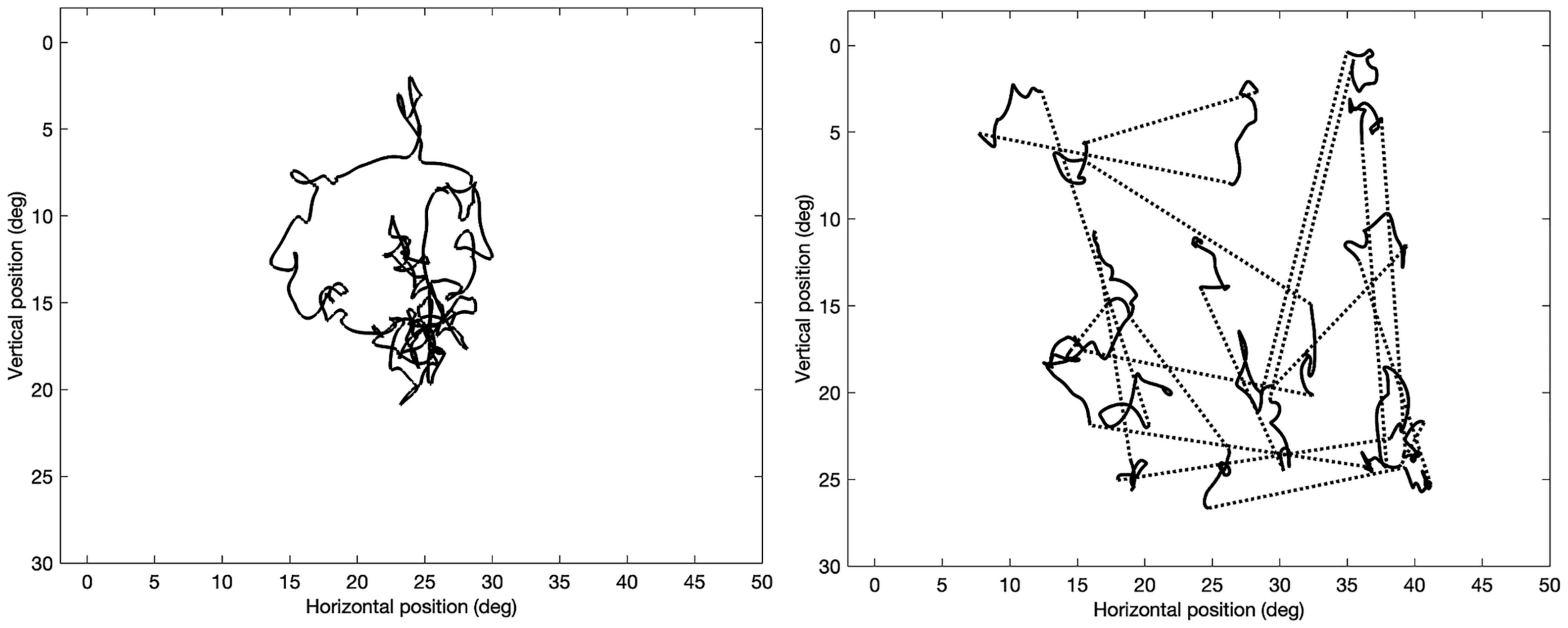
Example stimulus trajectories for a smooth pursuit trajectory (left) and saccadic pursuit trajectory (right). For the saccadic pursuit trajectory, the stimulus jumps are indicated by the dotted lines to note that the stimulus did not travel along these lines but simply disappeared in one location and reappeared in a new location. Note that each trajectory set consists of three saccadic pursuit trajectories, together sampling the entire visual field (Humphrey Field Analyzer 24–2 test locations).

The stimulus moved along a trajectory in both a smooth pursuit mode and a saccadic pursuit mode. During the smooth pursuit mode, the stimulus moves continuously, while both the direction and velocity of the motion vary pseudo-randomly. In the saccadic pursuit mode, the stimulus also moves continuously as in the smooth pursuit mode but additionally makes jumps to new test locations. A trajectory’s starting point was always the center of the screen. A trajectory set consisted of a single smooth and three saccadic pursuit mode trajectories (8) of 40 s each (see Figure 1).

For cases, the experiment consisted of one trajectory set (four trajectories of 40 s) with binocular measurement, followed by a different trajectory set with monocular measurement of the dominant eye. For both binocular and monocular controls, we used a corresponding subset of the original data for this study.

### 2.5 Pre-processing of eye movement data

Eye gaze positions were recorded in horizontal and vertical screen coordinates (pixels) and analyzed separately. During the experiment, the gaze and stimulus position vectors were stored together to ensure that they were time-linked, at the screen refresh rate of 240 Hz (a vector contains the position data corresponding to one trajectory). For this, the gaze position sampling rate was down sampled from 1,000 Hz (Eyelink sampling frequency) to 240 Hz to match the sampling rate of the stimulus. Sometimes, the gaze vector was slightly longer than the stimulus vector (between 0 and 2 frames, corresponding to a maximal timing error of 8.33 ms), likely due to small timing errors in either the Eyelink recording or the sending of the messages. For these vectors, we used the samples from the start of the gaze vector until the end of the stimulus vector length.

Before calculating the outcome measures, the raw gaze data were filtered for unreliable signal periods. These included (1) unrealistic peaks in velocity [>750 deg./s, not likely to be caused by actual eye movements (13, 14)] and (2) plateaus in position (zero velocity for at least two consecutive samples). The position plateaus were a consequence of how tracking failures (for example, due to blinking) were handled: those gaze positions were initially replaced by the last known gaze position rather than simply abandoned. Unreliable signal periods were set to Not a Number (NaN), including an additional 0.05 s of data preceding and following each unreliable signal period (to remove any additional noise surrounding blinks). If more than half of a trajectory had to be set to NaN, the whole trajectory was discarded. If this was a smooth pursuit trajectory, the whole smooth pursuit part of the set was excluded (as this was only a single trajectory). If two or more saccadic pursuit trajectories had to be discarded, the whole saccadic pursuit part of the set was excluded. Using this criterion, eight trajectories (2.6%) were discarded, and one case had to be excluded completely. Therefore, statistical analyses were performed on gaze data from 12 cases, 36 monocular controls, and 13 binocular controls.

### 2.6 Outcome measures

Three outcome measures were used. First, we defined tracking performance as the similarity between the stimulus and gaze positions over time. Second, we quantified the synchronization between the eyes as the positional difference between both eyes. Lastly, for the saccadic pursuit mode, we examined the dynamics of saccades in response to a stimulus jump. In all analyses, horizontal and vertical gaze positions were analyzed separately.

#### 2.6.1 Tracking performance

**Figure 2:**
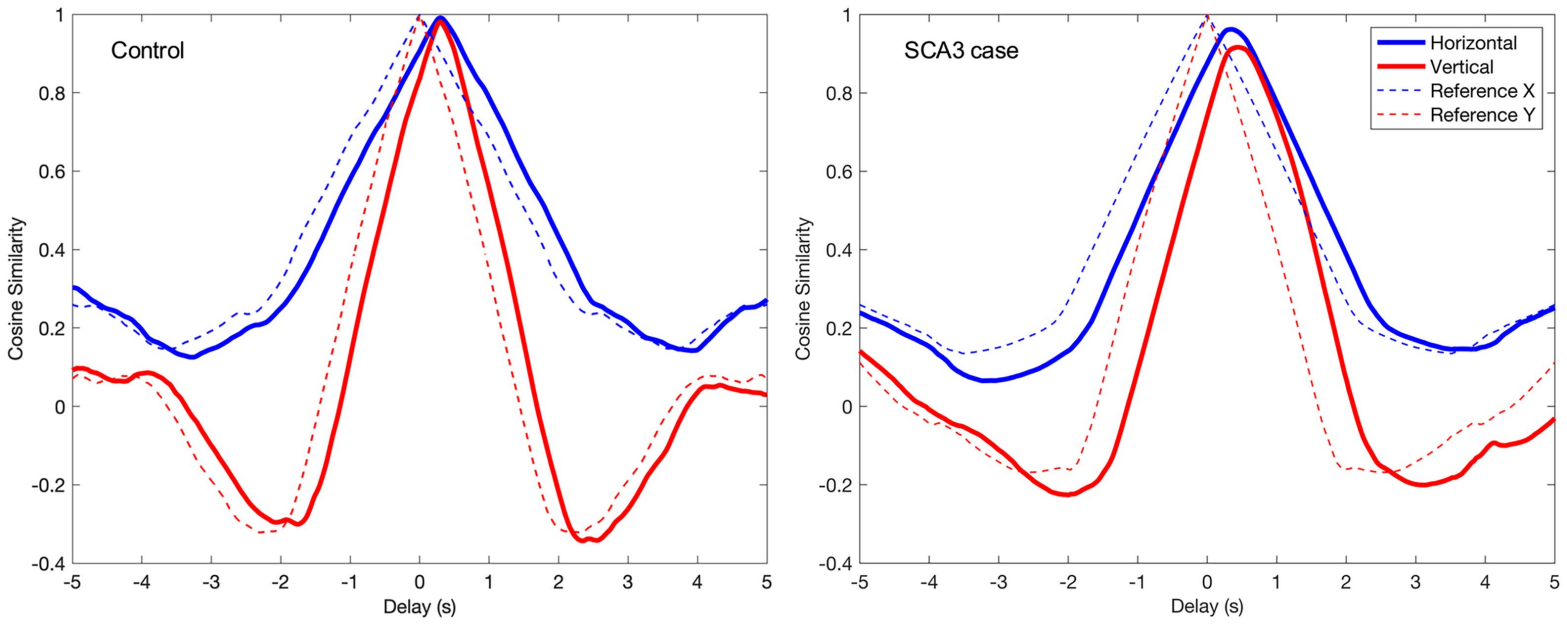
Example of the cosine similarity function for a single trajectory for a control (left) and a SCA3 case (right). The x-axis depicts the delay between stimulus and gaze in seconds; the y-axis depicts the cosine similarity. The dashed line (reference) shows the cosine similarity function for the stimulus with itself and therefore has a value of 1 at delay 0. The control’s cosine similarity function approximates the function of the reference, albeit with a small delay. The function of the SCA3 case is wider, with somewhat lower tracking performance and a larger delay.

Tracking performance was defined as the time-shifted cosine similarity between the stimulus and gaze vector. The time-shifted cosine similarity corrects for the inherent delay between gaze and stimulus movements [see also Vrijling and de Boer et al. (6)]. To do so, we calculated the cosine similarity for a series of delays between −5 and +5 s in steps of 0.0041 s (1 frame). The resulting output (see Figure 2 for an example) is a cosine similarity function. The tracking performance was then defined as the peak of this cosine similarity function.

The cosine similarity is the normalized inner product between the stimulus and gaze vector (Equation 1):


$$\text{Cosine Similarity}\;(A,B)=\frac{A\cdot B}{\left\|A\|\;\|B\right\|}$$ (1)

where *A* is the vector of stimulus positions over time and *B* the vector of gaze positions over time, ‖A‖ the Euclidean norm of vector *A*, and ‖B‖ the Euclidean norm of vector *B*. Values close to 0 indicate poor tracking performance and values near 1 very good tracking performance. We have previously shown that a tracking performance of around 0.98 for the smooth pursuit mode and around 0.91 for the saccadic pursuit mode can be considered ceiling level tracking performance, that is, these values, which lay close to the theoretical upper limit of 1.0, are the experimental upper limits for a healthy visual system (8).

For the overall tracking performance of a trajectory, we used the cosine similarity corresponding to the first positive peak of the cosine similarity function after 0 s (using the MATLAB function *findpeaks*). If no peak could be found (e.g., because tracking performance was very poor), the maximum cosine similarity between 0 and +3 s was used as the output value. For the three saccadic pursuit trajectories, we took the average tracking performance as the final outcome. For the binocular measurements, the tracking performance was determined separately for each eye and subsequently averaged.

#### 2.6.2 Synchronization between the eyes

For the binocular measurements only, we determined the synchronization between the eyes. To do so, we calculated the positional difference between the eyes by subtracting the gaze position of the left eye from the gaze position of the right eye at each time point. The positional differences were then averaged over the trajectory and, for the saccadic pursuit mode, subsequently averaged over the three trajectories to achieve the mean positional difference.

#### 2.6.3 Saccade dynamics

Lastly, for the saccadic pursuit mode only, we extracted the saccade characteristics from the Eyelink ASCII files (using the standard parser configuration with a velocity threshold of 30 deg./s and an acceleration threshold of 8,000 deg./s^2^) by separately storing from each saccade the start and end time (duration), the horizontal and vertical start and end positions (amplitude), and the velocity. All parameters were extracted from the end saccade messages (“ESACC”) in the ASCII files. We filtered out any erroneously recorded saccades using the following rules: exceeding an amplitude of 50 degrees (larger than the screen), amplitude smaller than 2 degrees (as these are unlikely to be in response to a stimulus jump), a duration shorter than 10 ms or longer than 150 ms, a velocity greater than 750 deg./s, and with a start or end position outside of the screen.

Saccades were separated into horizontal and vertical saccades based on the angle of the saccade; saccades with angles between 45 and 135 degrees or between 225 and 315 degrees were classified as vertical, the rest was classified as horizontal.

Lastly, we examined the saccadic main sequence. The saccadic main sequence describes the predictable relationship between saccade amplitude, duration, and peak velocity. Here, the main sequence was described via the relationship between saccade amplitude and saccade peak velocity to allow testing whether saccades were slower in cases.

### 2.7 Statistical analyses

All statistical analyses were conducted in R version 4.4.3 (15) using RStudio (version 2024.12.1 + 563). The study population was described using descriptive statistics [median with interquartile range (IQR)]. The differences in age and best-corrected visual acuity (BCVA; best eye for binocular measurements) between cases and controls were tested with a Mann–Whitney *U* test (using the *wilcox.test* function from the *stats* package, version 4.4.3). To determine whether cases differed from controls in tracking performance, absolute positional differences between the eyes, and median saccadic amplitude we performed linear regression separately for monocular and binocular gaze data, horizontal and vertical gaze data, and the different pursuit modes. In all models, group (cases, controls) was the main effect of interest. As cases differed from controls in age and BCVA (see section 3 below), we added these as covariates to all models. To correct for multiple hypothesis testing, *p*-values were FDR-adjusted (using the function *p.adjust* from the *stats* package), resulting in *q*-values. To determine whether the saccadic main sequence differed between cases and controls, we used a linear mixed effects regression model (using the *lmer* test from the *lme4* package, version 1.1–37) (16) that included saccade peak velocity as the dependent variable, saccade amplitude (centered on the mean), group, and viewing condition (binocular, monocular) as fixed effects, both as main effects and all interactions. Age and BCVA were added as covariates. Lastly, we included participant as a random intercept. The assumption of normality of the residuals was tested with the Shapiro–Wilk normality test (using the function *shapiro.test* from the *stats* package). The model violated the assumption of normality (W = 0.92, *p* < 0.001). Therefore, we used bootstrapping with 5,000 replications to determine the confidence intervals (CIs) of the model estimates (using the *confint* function from the *boot* package, version 1.3–31) (17, 18). Estimates for which the 95^th^ percentile CI did not include zero were considered significant.

## 3 Results

Table 1 shows the general characteristics of the study population. Monocular controls were somewhat older than the cases (*p* = 0.017), whereas the binocular controls were younger than the cases (*p* = 0.00013). Although the cases had somewhat worse visual acuity than both control groups (*p* = 0.0009 for the binocular measurements and *p* = 0.016 for the monocular measurements), they all had a normal visual acuity (all < 0.3 logMAR). All cases achieved binocular single vision with prism correction in primary gaze. However, they also all reported experiencing diplopia, mostly in lateral gaze. Note that the SONDA was performed without prism correction as this would have interfered with the eye-tracking.

**Table 1:** Characteristics of the study population.

	Cases (*n* = 13)		Monocular controls (*n* = 36)	Binocular controls (*n* = 13)	
Age, years; median (IQR)	60 (53, 70)		70 (67, 72)	23 (23, 30)	
Sex, female; n (%)	6 (46%)		16 (44%)	10 (77%)	
Eye tested^*^, OD; n (%)	7 (54%)		22 (61%)	N/A	
BCVA, logMAR; median (IQR)	OD: 0.05 (0, 0.10)	OS: 0.05 (0, 0.10)	−0.08 (−0.10, 0.01)	OD: −0.10 (−0.10, −0.10)	OS: −0.10 (−0.10, −0.10)
Age of disease onset, years; median (IQR)	42 (38, 45)		N/A	N/A	
Disease duration, years; median (IQR)	18 (15, 23)		N/A	N/A	
CAG repeats, number; median (IQR)	69 (67, 70)		N/A^**^	N/A^**^	

IQR, interquartile range; BCVA, best-corrected visual acuity; CAG, cytosine adenine guanine.

* For the cases this number relates to the monocular measurement.

** Not measured in these controls, but normal number of CAG repeats is 10–40 (28).

### 3.1 Tracking performance

**Figure 3:**
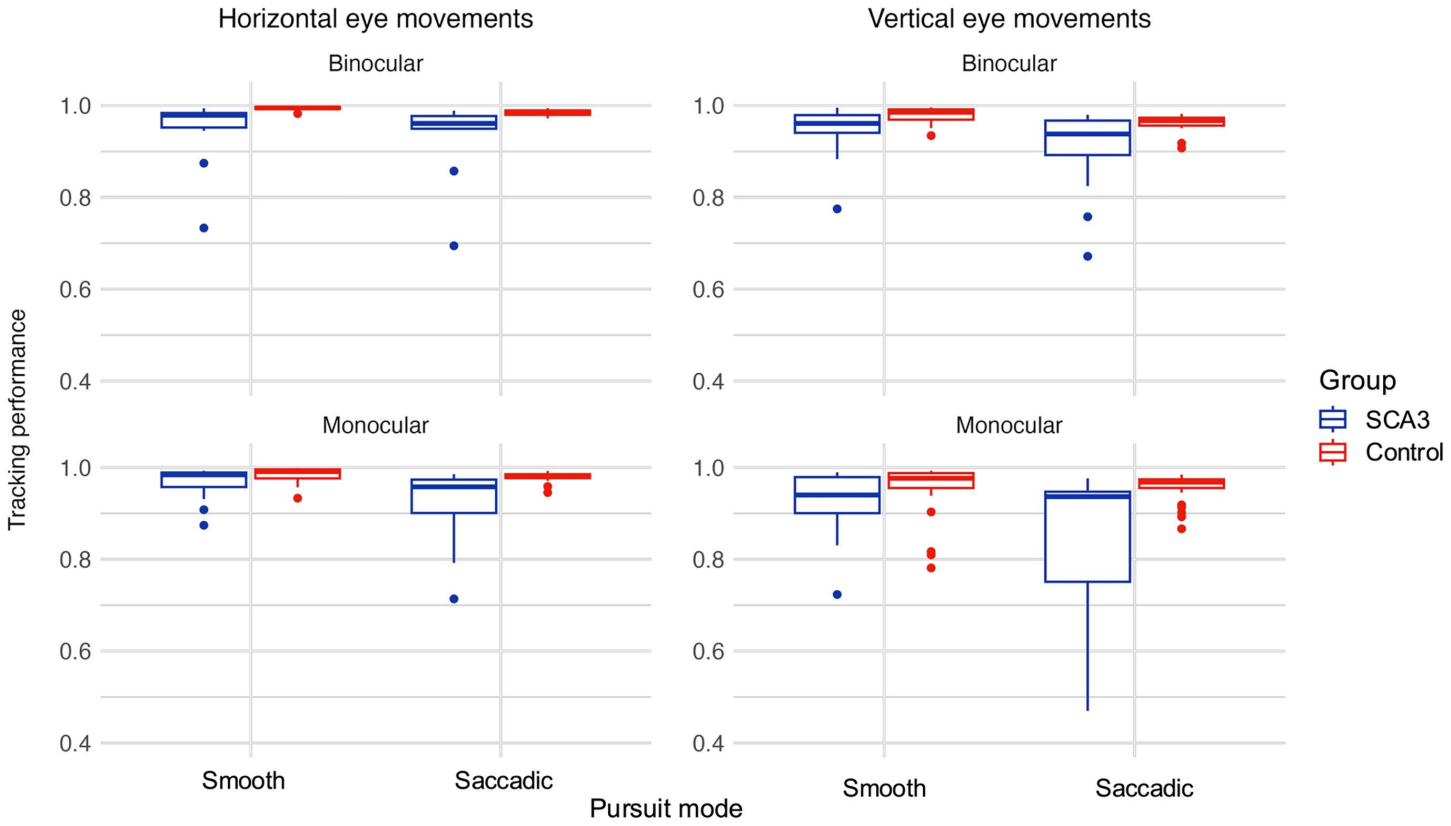
Tracking performance for horizontal (left) and vertical (right) eye movements for binocular (top) and monocular (bottom) viewing conditions.

Tracking performance in all groups and viewing conditions is shown in Figure 3. The figure clearly shows that most participants have a tracking performance at or near ceiling level, especially the controls. Many cases also reached ceiling level, but, on average, their tracking performance was slightly worse. However, this difference was only significant in the saccadic pursuit mode, monocular viewing condition. Participant age and visual acuity did not significantly affect tracking performance (all *q* values > 0.97 for age and > 0.47 for BCVA). All group comparisons are shown in Table 2.

**Table 2:** Median (SD) tracking performance for cases and controls in monocular and binocular viewing conditions, for smooth and saccadic pursuit modes.

	Cases	Controls	*p*-value	*q-*value
Smooth pursuit mode				
Binocular, horizontal	0.98 (0.075)	0.99 (0.004)	0.068	0.14
Binocular, vertical	0.96 (0.061)	0.99 (0.019)	0.25	0.33
Monocular, horizontal	0.98 (0.038)	0.99 (0.015)	0.027	0.071
Monocular, vertical	0.94 (0.075)	0.98 (0.052)	0.17	0.28
Saccadic pursuit mode				
Binocular, horizontal	0.96 (0.083)	0.99 (0.007)	0.29	0.32
Binocular, vertical	0.94 (0.098)	0.97 (0.022)	0.46	0.46
Monocular, horizontal	0.96 (0.086)	0.98 (0.009)	0.0017	**0.0070**
Monocular, vertical	0.94 (0.180)	0.97 (0.028)	0.0015	**0.0070**

Significant differences are indicated in bold.

### 3.2 Synchronization between the eyes

**Figure 4:**
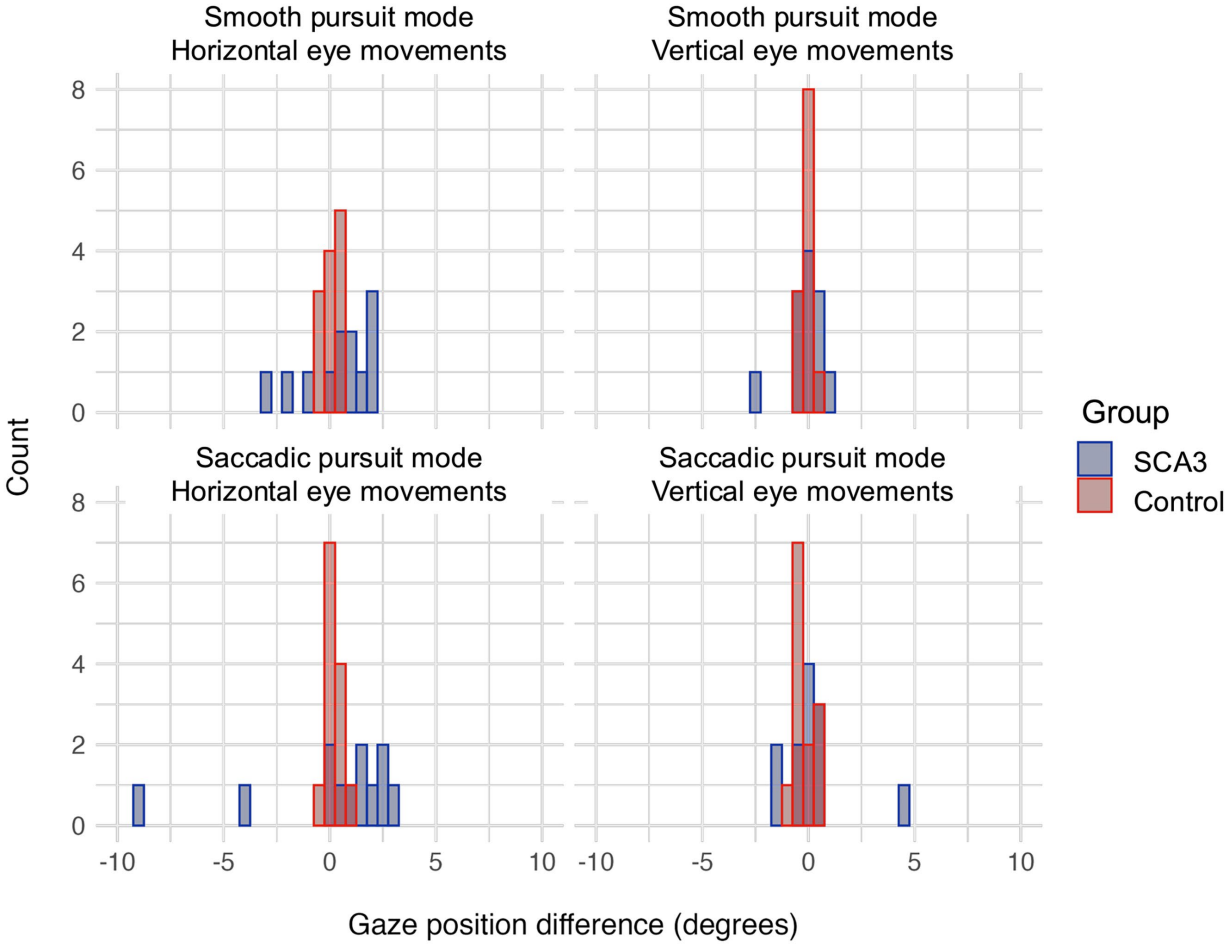
Histogram of positional differences between eyes (in degrees) for horizontal (left) and vertical (right) eye movements for both the smooth (top) and saccadic (bottom) pursuit modes. A positive difference indicates the gaze of OD was further to the left (for horizontal) or top (for vertical) of the screen than the gaze of OS.

Figure 4 shows a histogram of the positional difference between the eyes (averaged over trajectories) for cases and controls in the binocular viewing condition. When looking at the positional difference between the eyes, there were no differences in vertical eye movements for cases when compared to the binocular controls for both the smooth and saccadic pursuit modes. However, the horizontal positional difference between the eyes was larger in cases than controls, but only for the smooth pursuit mode. Participant age and visual acuity did not significantly affect positional differences between the eyes (all *q* values > 0.34 for age and > 0.23 for BCVA). Table 3 shows all group comparisons.

**Table 3:** Median (SD) absolute positional difference (in degrees) between the eyes for cases and controls in the binocular viewing condition.

	Cases	Controls	*p-*value	*q-*value
Smooth pursuit mode				
Horizontal	1.43 (0.78)	0.32 (0.13)	0.0078	**0.031**
Vertical	0.31 (0.69)	0.22 (0.20)	0.20	0.26
Saccadic pursuit mode				
Horizontal	1.75 (2.43)	0.23 (0.24)	0.056	0.11
Vertical	0.44 (1.27)	0.41 (0.28)	0.59	0.59

Significant differences are indicated in bold.

### 3.3 Saccade dynamics

**Figure 5:**
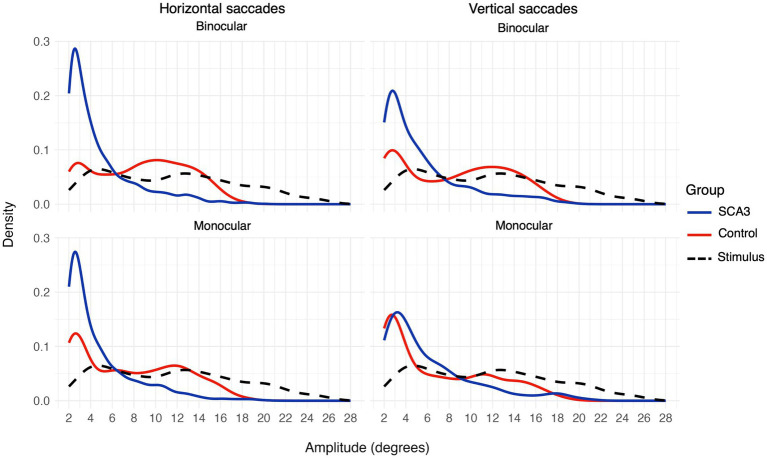
Saccadic amplitudes density plot for horizontal (left) and vertical (right) saccades or stimulus jumps (dashed black line) for binocular (top) and monocular (bottom) viewing.

**Figure 6:**
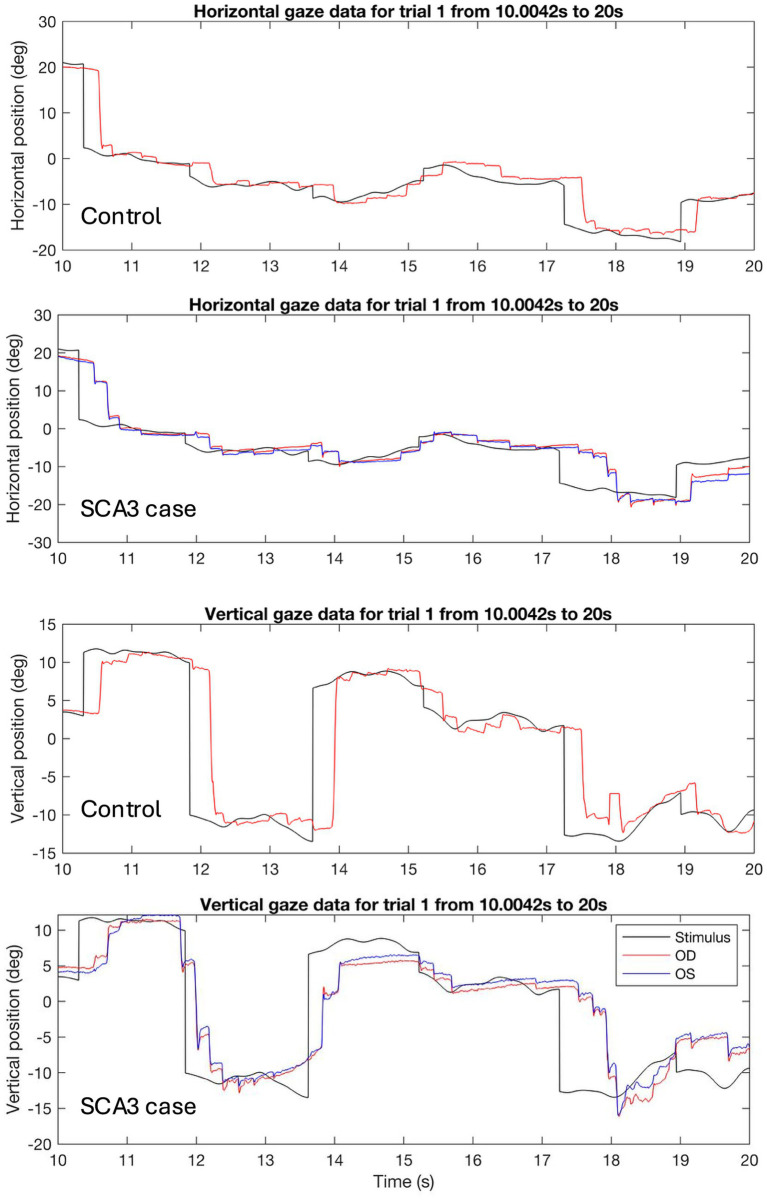
Example data showing saccades in response to three stimulus jumps during 10 s of the same trajectory for a control and a case. In the vertical gaze data (bottom 2 panels), it can be seen that the case made multiple small saccades in response to the stimulus jumps around 12, 14, and 17 s, whereas the control made a single large saccade, sometimes followed by a small corrective saccade. The same saccadic behavior can be seen in the horizontal gaze data (top two panels) around 17 s for the case.

When looking at the saccadic amplitudes of the eye movements (see Figure 5 and Table 4) there was a remarkable difference between cases and controls; cases made mostly small saccades, whereas the amplitude distribution of controls’ saccades more closely followed the distribution of the stimulus jump amplitudes. This was not the case for the vertical saccades. Here, the difference in median saccadic amplitude between controls and cases was not significant (see Table 4). Participant age and visual acuity did not significantly affect saccadic amplitudes (all *q* values > 0.84 for age and > 0.23 for BCVA) Individual differences were present however, especially in the group of cases (see Supplementary Figures S1–S4). The raw gaze data (see Figure 6 for an example) revealed that cases made several small saccadic movements to reach the stimulus, whereas controls mostly reached the stimulus with a single saccade and a smaller corrective saccade.

**Table 4:** Median (SD) saccadic amplitudes (in degrees) for cases and controls in monocular and binocular viewing conditions.

	Cases	Controls	*p*-value	*q*-value
Binocular, horizontal	3.65 (3.27)	9.00 (4.22)	0.0053	**0.011**
Binocular, vertical	4.24 (3.79)	8.88 (4.80)	0.049	0.066
Monocular, horizontal	3.49 (3.25)	7.19 (4.65)	<0.001	**<0.001**
Monocular, vertical	4.54 (4.19)	5.07 (4.75)	0.21	0.21

Significant differences are indicated in bold.

**Figure 7:**
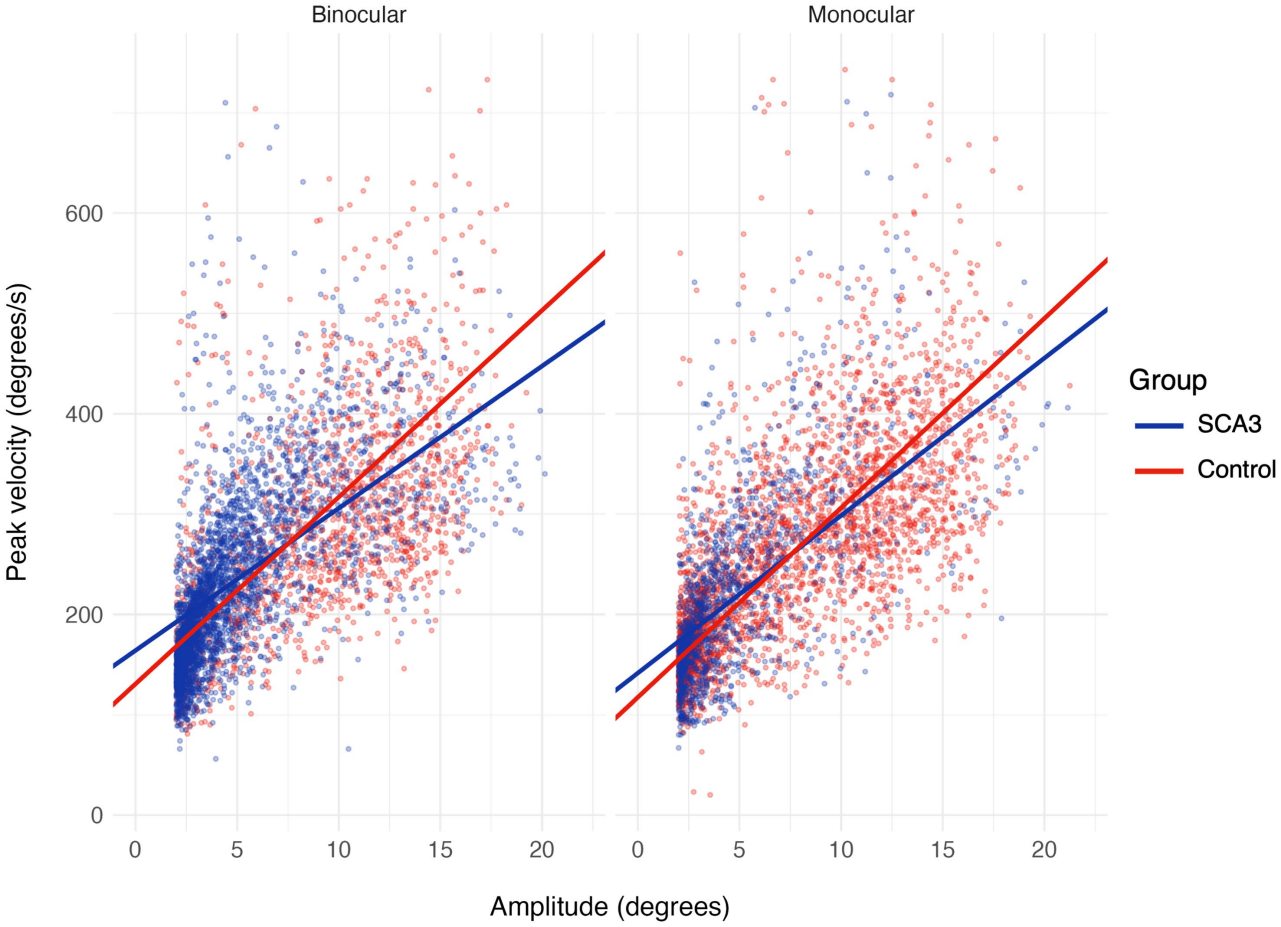
Saccadic main sequence (peak velocity as a function of amplitude) for the binocular (left) and monocular (right) viewing condition in the saccadic pursuit mode.

There was no evidence for a reduced velocity of saccades in the cases. The regression model showed that the slope of the main sequence (see Figure 7) was 14.2 deg./s per 1 degree increase in amplitude for the binocular controls [estimate (95% CI) = 14.2 (13.4, 14.9)] and 4.4 (3.4, 5.4) deg./s steeper for the cases, giving a total slope of 18.6 deg./s. The model further showed a peak velocity of 237 (140, 335) deg./s at the average saccade amplitude of 6.5 deg., and a slight increase in the slope for monocular controls [estimate (95% CI) = 1.5 (0.6, 2.4)]. Other effects were not significant (95% CI includes 0).

## 4 Discussion

The present study aimed at identifying oculomotor abnormalities in SCA3 patients that could be readily detected using eye-tracking during SONDA. Additionally, we aimed to find eye-tracking based parameters that could distinguish cases from controls that could potentially serve as biomarkers in the monitoring of oculomotor abnormalities in SCA3. Overall, we found that while patients with SCA3 are well capable of following a moving stimulus, indicated by their high overall tracking performance, they perform the task differently compared to the controls. More specifically, their eyes are not synchronized in their movements, indicated by a larger positional difference between their eyes, and they make many small saccades in response to a stimulus jump. Interestingly, often the differences were only significant for horizontal, but not vertical eye movements. Below, we discuss these findings in more detail.

### 4.1 Good tracking performance in SCA3 patients

In the saccadic pursuit mode, cases showed significantly lower tracking performance compared to monocular controls. Differences were non-significant for the binocular viewing condition and in the smooth pursuit mode. Despite these differences, cases showed high tracking performance. For example, cases’ tracking performance was higher than what we have seen previously in those with moderate or advanced glaucoma (6). Therefore, even though the cases indicated verbally they had difficulties performing the task, they were still able to exhibit good tracking of the stimulus. This finding additionally highlights that while tracking performance may be a good outcome parameter when screening for visual field defects, as an outcome parameter it is not necessarily well suited to screen for oculomotor disorders. Therefore, when screening for visual impairment through SONDA with any (unknown) cause, it is important to always consider multiple outcome parameters.

### 4.2 Eye movements are affected in SCA3 patients, but only in the horizontal direction

Looking at other parameters, namely the synchronization between the eyes (calculated as the positional difference between the eyes), we found that eye movements were affected in cases. Interestingly, only horizontal eye movements showed increased positional difference in cases compared to controls, and only in the smooth pursuit mode. As horizontal strabismus patterns are more common than vertical ones (3, 19, 20), this finding is not surprising. However, another study found abnormalities in both horizontal and vertical eye movements, with a tendency for especially vertical saccades to be abnormal compared to controls (21). This difference might be related to the fact that the cases in the Wu et al. study (21) were in a relatively mild stage of the disease (with most still able to walk independently and a mean disease duration of 4.5 years) as well as the nature of the stimulus used: the stimulus always moved only in the four cardinal directions (up, down, left, right), in a predictable fashion (e.g., fixed amplitude), and movement was trial-based. In SONDA, the stimulus movements are pseudo-random, continuous, and always have both a vertical and horizontal component. However, further studies are necessary to uncover whether such differences in the stimulus can explain the differences in findings.

### 4.3 Saccadic amplitude distributions as a biomarker for diplopia

Group analysis showed that saccadic amplitude distributions were different for cases as compared to controls, with cases producing mostly small, and thus hypometric saccades, whereas the saccades of controls more closely followed the stimulus jump amplitude distribution. Similar to our findings for between-eye synchronization, the difference in saccadic amplitude was only significant for horizontal saccades. In the literature, both hypermetric (22) and hypometric saccades (23) have been described. It has been suggested (24) that this disagreement can be explained by the clinical stage of the included cases; most cases in Rivaud-Pechoux and colleagues’ study (23) were in a moderate-to-severe stage whereas those in Buttner et al.’s study (22) were mostly early stage. In line with this, inspecting saccadic amplitude distributions of individual cases (see Supplementary Figures S1, S3) showed that there were large individual differences and these differences seemed related to the severity of oculomotor disorder. Three cases experienced significant difficulty in fusing the images from both eyes and experienced persistent diplopia; these cases also exhibited the most pronounced deviations from control participants in their saccadic amplitude distributions. In contrast, one case had minimal oculomotor symptoms and demonstrated a saccadic amplitude distribution comparable to that of the controls. It is good to note that while these oculomotor abnormalities may not directly reflect the ability of the fusional control of an individual, they do reflect the status of the oculomotor control in broader aspect. None of the participants showed clear evidence for hypermetric saccades, at least not as indicated by their saccadic amplitude distributions, suggesting that saccade gain abnormality is not only related to clinical stage. Relatedly, Alexandre and colleagues (25) found both hyper- and hypometric saccades in SCA3 cases, and this seemed unrelated to their clinical stage as assessed with the SARA (Scale for the Assessment and Rating of Ataxia) (26). Based on our findings, we hypothesize that the distribution of saccadic amplitudes in response to stimulus jumps could potentially be a biomarker for diplopia. Further research in larger and more diverse patient cohorts is required to determine whether saccadic amplitude distributions can reliably differentiate levels of disease severity and predict the likelihood of successful intervention for diplopia.

### 4.4 Limitations

This study included 13 participants with SCA3. A large sample size given the rarity of the disease and the fact that participating is quite demanding for these patients, but a too small sample size for such a heterogeneous disorder to draw robust conclusions regarding the validity of the suggested biomarkers. Therefore, further research with more participants and a longitudinal study design is needed to confirm the proposed biomarkers.

One limitation of the current version of SONDA is that it lacks the ability to test fixation stability. Fixation abnormalities and gaze-evoked nystagmus are commonly found in SCA3 and are proposed to help distinguish SCA3 from other spinocerebellar ataxias (4, 21, 23, 25, 27). Future studies could adapt the SONDA test to additionally include periods with a stable stimulus position. While this would increase test-time, it might contribute to other biomarkers, such as quantification of fixation drift and microsaccades, for diplopia severity in SCA3 and thus improve treatment strategies.

### 4.5 Conclusion

The present study shows that SONDA can give meaningful information on how eye movements are affected in SCA3 and quantify the amount of disorder. It has additionally provided potential biomarkers for disease severity in the form of the shape of the saccadic amplitude distribution and the magnitude of the positional difference between the eyes. These findings pave the way for diagnostic applications of SONDA to inform treatment strategies for SCA3, although more work is needed to determine how these potential biomarkers could be practically implemented in the clinic. The logical next steps are studies with more participants and longitudinal data collection to confirm the validity of the suggested biomarkers and to define thresholds based on normative control datasets.

## Data Availability

The datasets presented in this study can be found in online repositories. The names of the repository/repositories and accession number(s) can be found below: https://doi.org/10.34894/BUVAJM.
