# Supplementary material for: Continuous visual stimulus tracking to quantify eye motility in spinocerebellar ataxia type 3
Source: Front Neurol. 2025 Dec 15;16:1650269. doi: 10.3389/fneur.2025.1650269 (PMC12745225; doi:10.3389/fneur.2025.1650269)
Supplement: Supplementary file 1 [file Data_Sheet_1.pdf]

Supplementary material

Continuous visual stimulus tracking to quantify eye motility in spinocerebellar ataxia type 3

M.J. de Boer[1*], R.A. Wasmann[2*], J.W.R. Pott[2], F.W. Cornelissen[1], N.M. Jansonius[1,2]

Corresponding author: Minke de Boer. Email: minke.de.boer@rug.nl

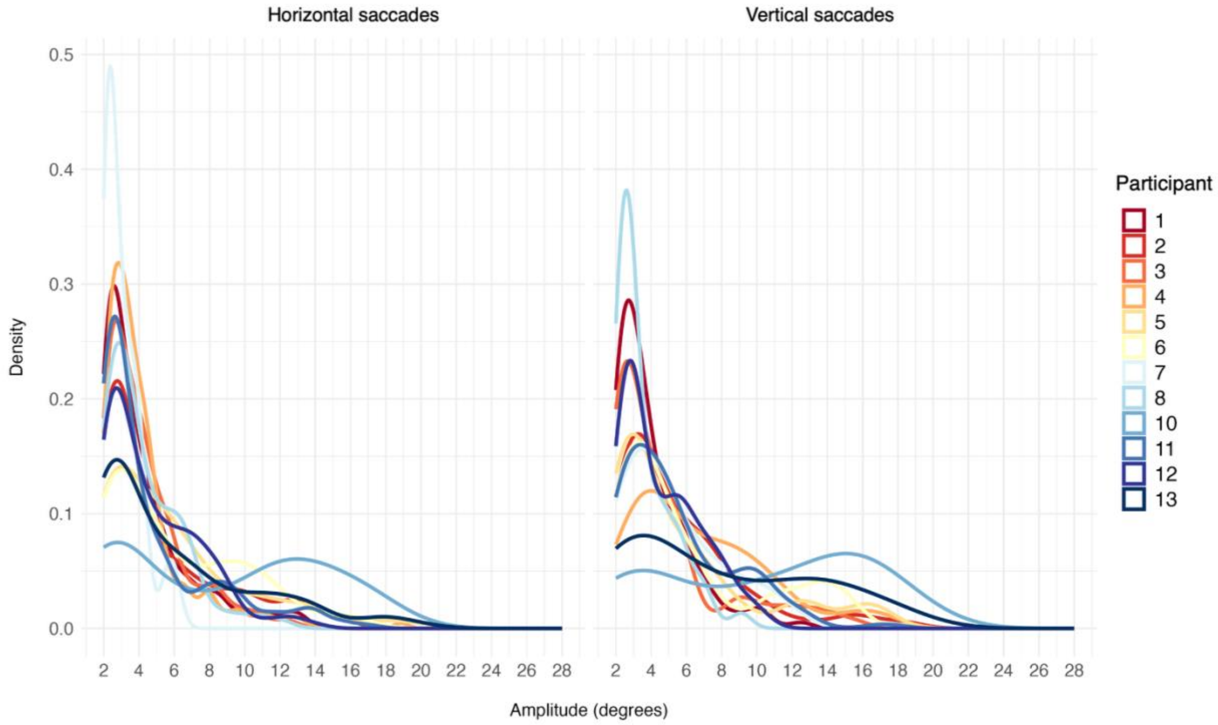

Figure S1. Saccadic amplitudes density plot for individual cases for horizontal (left) and vertical (right) saccades for binocular viewing.

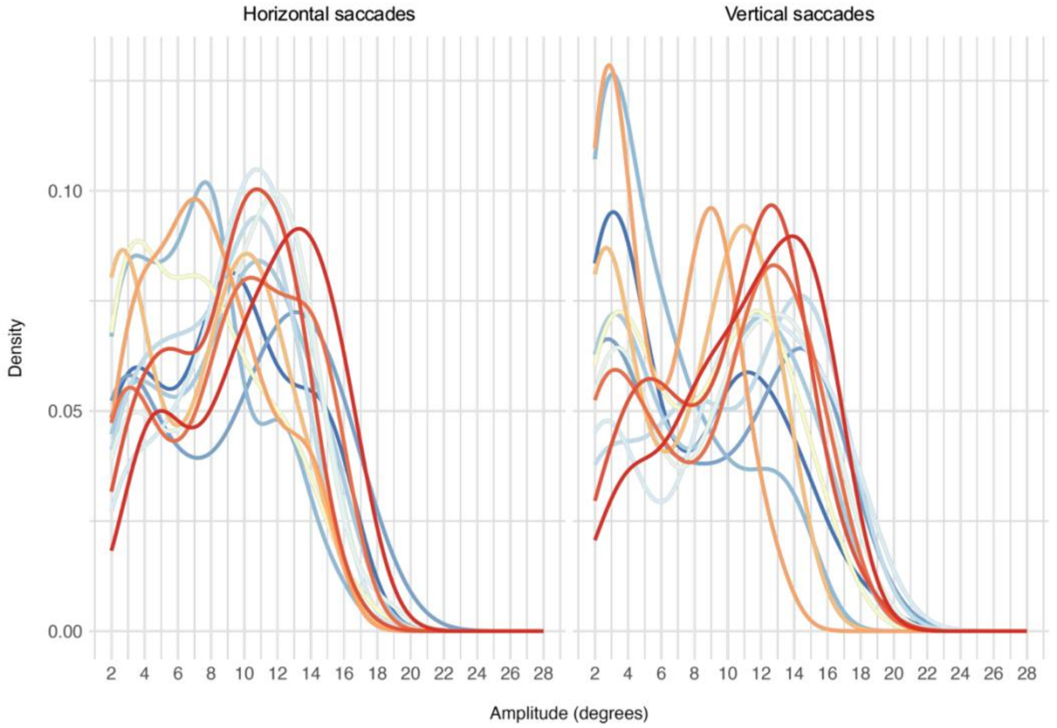

Figure S2. Saccadic amplitudes density plot for individual controls for horizontal (left) and vertical (right) saccades for binocular viewing.

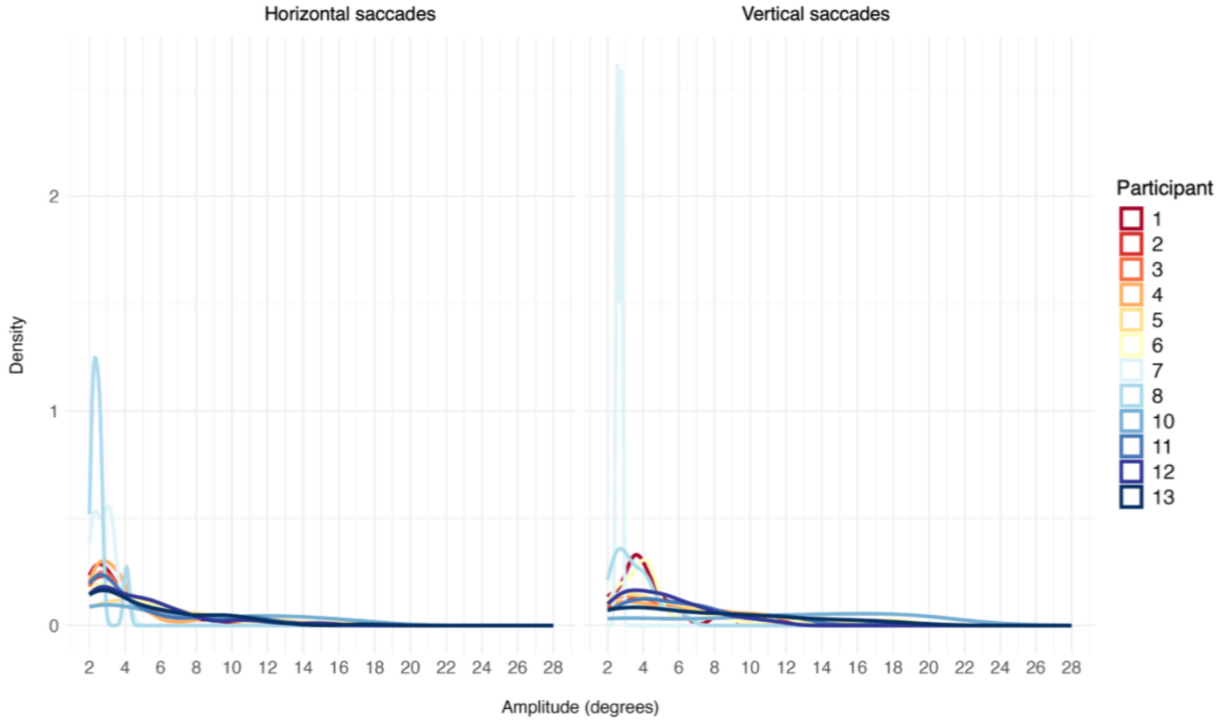

Figure S3. Saccadic amplitudes density plot for individual cases for horizontal (left) and vertical (right) saccades for monocular viewing.

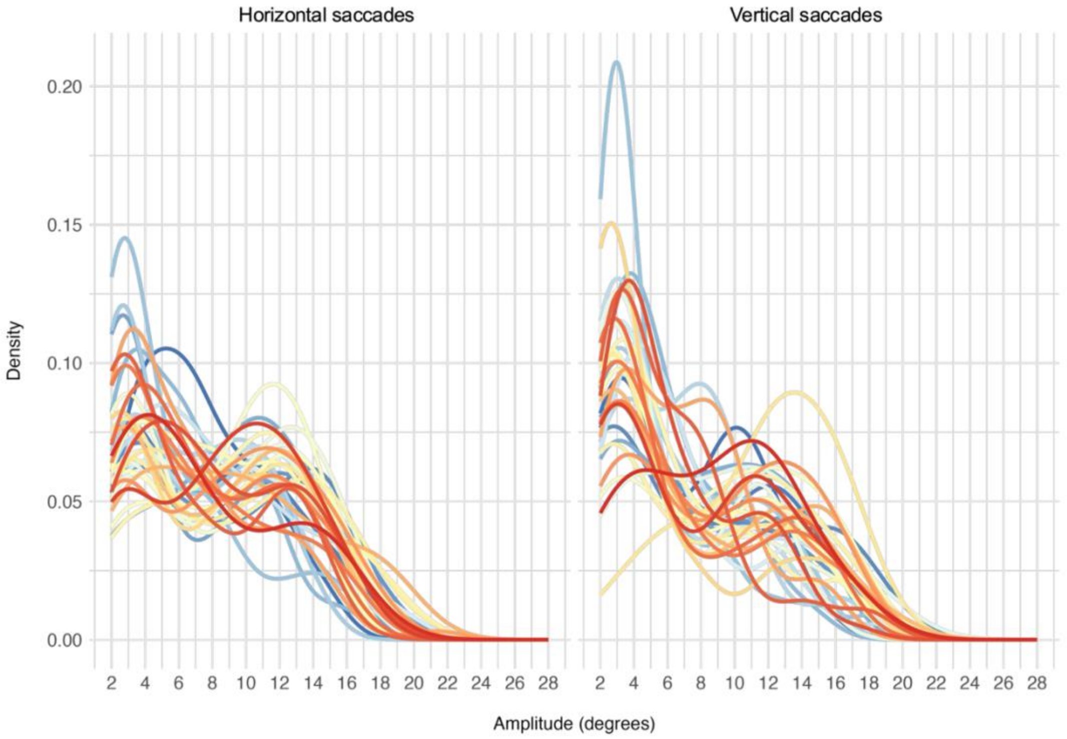

Figure S4. Saccadic amplitudes density plot for individual controls for horizontal (left) and vertical (right) saccades for monocular viewing.
